# Submicroscopic *Plasmodium falciparum* Carriage and Molecular Markers of Antimalarial Drug Resistance Among Outpatients Attending Korle Bu Teaching Hospital, Ghana

**DOI:** 10.3390/tropicalmed11070190

**Published:** 2026-07-09

**Authors:** Benjamin Tetteh Mensah, Hannah Otu, Dorinda Naa Okailey Armah, Comfort Teiko Abuanor, Lucas Amenga-Etego, Samuel Antwi-Baffour

**Affiliations:** 1 Department of Medical Laboratory Sciences, School of Biomedical and Allied Health Sciences, College of Health Sciences, University of Ghana, Legon, Accra P.O. Box LG25, Ghana; mykroworld@gmail.com; 2 The Central Laboratory, Korle-Bu Teaching Hospital, Korle-Bu, Accra P.O. Box KB77, Ghana; kukuaakrong@gmail.com; 3 Department of Occupational Therapy Sciences, School of Biomedical and Allied Health Sciences, College of Health Sciences, University of Ghana, Legon, Accra P.O. Box LG25, Ghana; dnoarmah@ug.edu.gh; 4 Department of Gene Therapy, Thrivus University for Biomedical Science and Technology, Constellation Avenue, Lashibi, Accra P.O. Box LB36, Ghana; cabuanor@gmail.com; 5 West African Centre for Cell Biology of Infectious Pathogens, Department of Biochemistry, Cell and Molecular Biology, University of Ghana, Legon, Accra P.O. Box LG25, Ghana; lamengaetego@ug.edu.gh

**Keywords:** *Plasmodium falciparum*, submicroscopic malaria, antimalarial drug resistance, molecular markers, LAMP-PCR, Oxford Nanopore sequencing, malaria surveillance

## Abstract

**Background:** Malaria remains a major public health challenge in sub-Saharan Africa, where asymptomatic infections continue to hinder elimination efforts. Although the clinical and epidemiological consequences of microscopic infections are well documented, the health implications of submicroscopic *Plasmodium falciparum* infections remain poorly understood, particularly in Ghana. This study assessed the burden of submicroscopic *P. falciparum* infections and their association with antimalarial drug resistance markers among outpatients attending Korle Bu Teaching Hospital. **Methods:** A cross-sectional study involving 345 participants was conducted. Malaria infection was assessed using mRDT, blood smear microscopy, and LAMP-PCR for parasite detection and species identification. Antimalarial drug resistance markers were analyzed by multiplex PCR and Oxford Nanopore sequencing. Statistical analysis was performed using STATA version 14, applying Pearson’s chi-square test and logistic regression. **Results:** The overall prevalence of asymptomatic *P. falciparum* infection was 35.0% (117/334), with 18.9% microscopic and 16.1% submicroscopic infections. The wild-type *pfcrt* K76 allele, associated with chloroquine susceptibility, was highly prevalent (90.1%). **Conclusions:** Asymptomatic *P. falciparum* infections are common in this population, with a substantial proportion occurring at submicroscopic levels. The high prevalence of chloroquine-susceptible parasites and identified transmission hotspots underscore the need for sensitive diagnostics and targeted interventions to support malaria elimination efforts in Ghana.

## 1. Introduction

Malaria remains a major global health burden, particularly in sub-Saharan Africa, despite sustained control efforts, with asymptomatic and submicroscopic infections continuing to drive transmission [1,2]. In highly endemic settings, over 90% of infections may be asymptomatic, allowing undetected carriers to sustain parasite transmission [3,4]. Traditionally defined as microscopy-detected parasitemia in the absence of symptoms [5], asymptomatic malaria also includes low-density infections detectable only by PCR, with evidence showing that up to two-thirds of microscopy-negative individuals harbor submicroscopic infections [6].

Beyond their epidemiological importance, submicroscopic infections are associated with chronic mild anemia and alterations in red blood cell (RBC) indices such as MCV, MCH, and RDW, reflecting ongoing erythrocyte destruction [7,8]. Persistent low-density parasitemia may also promote antimalarial drug resistance under sub-therapeutic drug pressure [9]. Key *P. falciparum* resistance genes: *pfcrt*, *pfdhfr*, *pfdhps*, *pfmdr1*, and *pfkelch13* mediate resistance to major antimalarial drugs [10,11], yet their prevalence in asymptomatic infections in Ghana remains poorly defined. Asymptomatic malaria has additionally been linked to anemia and impaired cognitive performance [5,7,8], and recent studies in Ghana report increasing rates of asymptomatic and submicroscopic infections across transmission zones [12,13].

Given these challenges, a comprehensive understanding of the epidemiology of submicroscopic infections, and associated resistance profiles is urgently needed. This study therefore aims to determine the prevalence of submicroscopic *P. falciparum* infections and assess associations with molecular markers of antimalarial drug resistance to inform malaria elimination strategies in Ghana.

## 2. Materials and Methods

### 2.1. Study Setting

The study was carried out at the Central Laboratory and Child Health Laboratory of the Korle-Bu Teaching Hospital, located in the Ablekuma South Municipal Assembly, a sub-district in the Greater Accra Region.

### 2.2. Study Design

A cross-sectional study was conducted.

### 2.3. Study Population

Patients from the Outpatient Department (OPD) who visited the Central laboratory and the Child health laboratory comprised the study population.

#### 2.3.1. Inclusion Criteria

This study included afebrile patients aged 2 years or older who had no signs or symptoms of malaria and had not taken any artemisinin-based combination therapies (ACTs) in the preceding 2 weeks, after providing informed consent. We also recorded any recent symptoms, prior history of fever, or use of antipyretics. Participants who tested negative for malaria parasites were assigned to the control group.

#### 2.3.2. Exclusion Criteria

1. Patients with signs and symptoms of malaria (defined as axillary temperature above 37.5 °C) at the time of recruitment.
2. Patients who had taken antimalarial drugs (ACTs) within the past two weeks and
3. Patients who are severely ill, pregnant women and patients with hemoglobinopathies.

### 2.4. Sampling Method

Patients from the Outpatient Department (OPD) of the Central Laboratory and the Child Health Laboratory of the Korle Bu Teaching Hospital were recruited into the study using purposive sampling technique to deliberately select participants who were considered most appropriate for addressing the study objectives.

### 2.5. Study Procedure

#### 2.5.1. Enrolment and Clinical Groups

Data was collected between April and July 2025. A total of 345 participants were enrolled after passing the inclusion criteria. Participants were then interviewed using a structured questionnaire with information on age, sex, location, number of household members, occupation, educational level, history of fever, number of previous episodes of malaria/fever, last date of malaria diagnosis and result of diagnosis and antimalarial drugs used in malaria treatment. Participants were then grouped into three distinct age categories, namely, less than 18 years, 18 to 35 years and 36 and above, classified as children, young adult and older adult populations. During the study, four samples were lost due to logistical mishaps, and seven samples were excluded from the study following review of the full blood count results, which revealed marked haematological abnormalities, including markedly elevated white blood cell counts and severe anaemia (very low haemoglobin concentrations). These abnormalities may indicate underlying infections, inflammatory conditions, haematological disorders, or other comorbidities that could influence the study variables and introduce bias into the analysis. Excluding these samples helped to ensure a more homogeneous study population and improved the validity of the findings. The resultant 334 samples remaining were used for the analysis.

##### Definition of Clinical Groups

Asymptomatic malaria infections were defined and categorized into three clinical groups according to the microscopy and PCR results. Samples which tested positive for microscopic asymptomatic infections, were defined as positive microscopy infections. Also, submicroscopic infections were defined as negative microscopy but positive LAMP results, with uninfected, defined as negative for both microscopy and LAMP.

##### Sample Collection and Other Laboratory Procedures

Approximately 3 mL of venous blood was collected aseptically into 5 mL EDTA tubes and gently mixed to prevent clotting. Each sample was labelled with a unique participant identification code to ensure confidentiality. Aliquots of the blood were used for multiple analyses: dried blood spots (DBS) were prepared by spotting 200 µL onto 3 mm Whatman No. 1 filter paper (GE Healthcare Life Sciences, Little Chalfont, Buckinghamshire, UK) for subsequent molecular work, while 6 µL and 2 µL were used to prepare thick and thin blood films on labelled glass slides. These films were stained with 10% Giemsa following standard procedures. Thin films were fixed in absolute methanol while thick films were lysed prior to staining. An additional 5 µL of blood was used for malaria rapid diagnostic testing using the *OnSite* Malaria Rapid Diagnostic Test Kit, which detects *P. falciparum* (HRP-II) and pan-*Plasmodium* aldolase antigens for species differentiation. The DBS samples were air-dried, stored in zip-lock bags with desiccants, and transported to the malaria laboratory of West African Centre for Cell Biology of Infectious Pathogens (WACCBIP) for molecular analysis.

Microscopy was performed using both thick and thin blood films. The thick films were used for parasite detection and quantification and thin films for species identification. Slides were examined under ×100 oil immersion, with at least 100 high-power fields reviewed before declaring a sample negative. Parasite density was estimated by counting asexual parasites per 200 white blood cells and multiplying by an assumed leukocyte count of 8000/µL [14]. All slides were independently read by two experienced microscopists blinded to RDT results and to each other’s readings; discrepancies were resolved by a third reader, and the average of the closest counts was recorded. A slide was considered positive if at least one parasite was detected, with additional fields examined to identify mixed infections where necessary.

For molecular analysis, genomic DNA was extracted from DBS samples (both microscopy-positive and -negative) using the QIAamp DNA Blood Mini Kit (Qiagen, Hilden, North Rhine-Westphalia, Germany), with minor modifications to optimize yield. Briefly, 2–3 mm DBS punches were lysed using buffer ATL and Proteinase K, followed by incubation, binding, washing, and elution steps according to the manufacturer’s protocol. DNA was eluted in 50 µL buffer AE and stored at −20 °C. Quantification was performed using the Qubit dsDNA High Sensitivity Assay Kit on a Qubit fluorometer (Thermo Fisher Scientific, Waltham, MA, USA).

**Figure 1:**
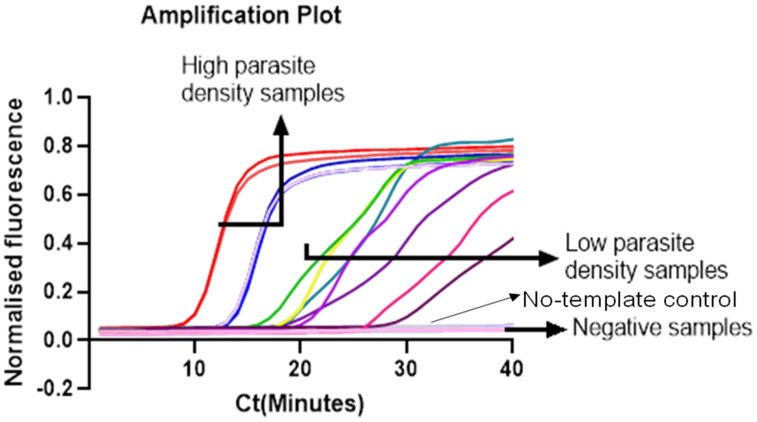
Amplification plots after LAMP assay analysis.

**Figure 2:**
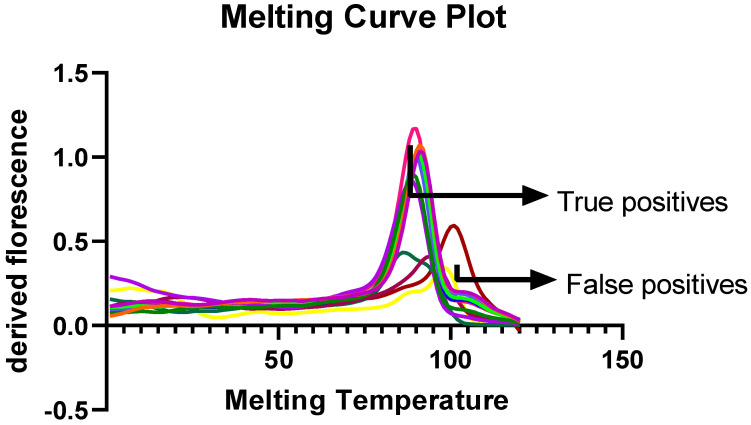
Melting curve plots depicting true positives and false negatives after LAMP assay analysis.

To detect submicroscopic malaria infections, all microscopy-negative samples were further analysed using a real-time loop-mediated isothermal amplification (LAMP) assay targeting *P. falciparum*, *P. ovale*, and *P. malariae* (Figure 1) [15,16]. Separate species-specific LAMP reactions were prepared in a final volume of 20 µL containing 1× isothermal amplification buffer, MgCl_2_ (8 mM), betaine (0.8 M), 1.4 mM each of dATP, dCTP, and dGTP, 1.4 mM dUTP, species-specific primer sets comprising outer primers (F3 and B3, 0.2 µM each), inner primers (FIP and BIP, 1.6 µM each), and loop primers (LF and LB, 0.8 µM each), 8 U of Bst DNA polymerase large fragment (Bst-LF), 1 µM SYTO-9 fluorescent dye, and 3 µL of extracted DNA template (Supplementary Materials). The remaining volume was adjusted with nuclease-free water. For each assay run, a positive control (DNA from a confirmed Plasmodium-positive sample) and a no-template negative control were included. Amplification was performed on the QuantStudio™ 5 Real-Time PCR System (Applied Biosystems, Foster City, CA, USA) at a constant temperature of 65 °C for 30 min, followed by a melt curve analysis from 65 °C to 95 °C with fluorescence acquisition at 0.1 °C increments. Samples were considered positive when they demonstrated a characteristic amplification curve and a species-specific melting temperature (Tm) consistent with that of the corresponding positive control (Figure 2).

#### 2.5.2. Detection of Drug-Resistant Molecular Markers

##### Multiplex PCR (Panel Amplification) and Gel Electrophoresis

**Figure 3:**
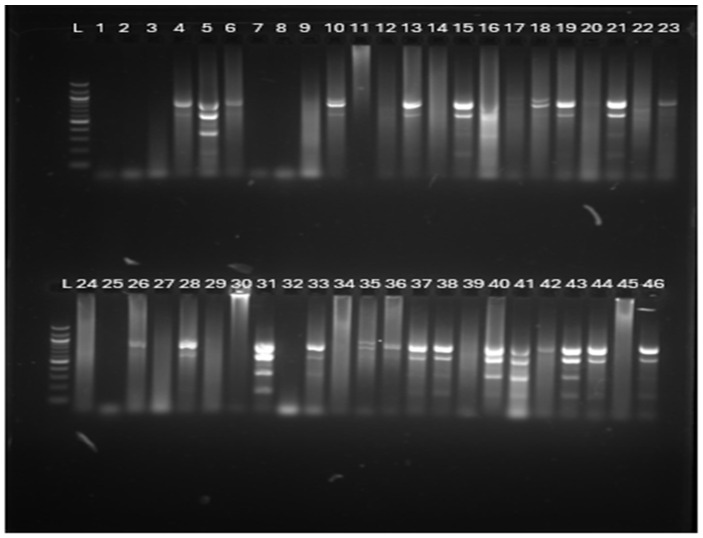
Gel electrophoresis showing Multiplex PCR amplification of *P. falciparum* drug resistance markers.

All malaria-positive cases, that is, both microscopic and submicroscopic malaria cases, were subjected to antimalarial drug resistance analysis to identify molecular markers of drug-resistant parasite strains. Multiplex PCR was performed to amplify fragments of the *Plasmodium falciparum* genes *pfcrt*, *pfdhfr*, *pfdhps*, *pfmdr1*, and *pfkelch13*, following the method described previously [15,16] (Supplementary Materials). Reactions were prepared in 96-well PCR plates by combining 47 µL of pre-prepared PCR master mix with 2 µL of high-quality extracted genomic DNA (~5 ng/µL, final input ~10 ng). The contents were mixed thoroughly by gentle pipetting. A no-template negative control (using nuclease-free water in place of DNA) and a positive control (genomic DNA from the *P. falciparum* reference isolate KH02) were included in each run. Plates were sealed tightly and briefly centrifuged to collect all liquid at the bottom of the wells before amplification. Thermocycling was carried out using a PCRmax™ Alpha Cycler 4 (Thermo Fisher Scientific, USA) under already set thermocycling conditions [15]. Amplification products were resolved by electrophoresis on 2% agarose gels and visualized using an Amersham Imager 600 (Cytiva, Tokyo, Japan) to confirm the presence of fragments of the expected size (Figure 3).

##### Amplicon Purification and Library Preparation

PCR amplicons were purified using the QIAamp^®^ DNA MiniElute Kit (Qiagen, Germany) according to the manufacturer’s instructions. Eluted gDNA amplicons were then quantified as described above and stored at 4 °C. Afterwards, three sequencing libraries were prepared for the purified PCR amplicons (22 clinical isolates, KH02 positive control and negative control per batch) using the SQK-NBD112.24 native barcoding kit (Oxford Nanopore Technologies, Oxford, UK). The manufacturer’s protocol was followed for the library preparation. Briefly, 12.5 μL of each purified DNA amplicon (~200 fmol) was end-prepped using 1× Ultra II End-prep enzyme mix, incubated for 5 min at 20 °C and 5 min at 65 °C. End-prepped PCR amplicons were then purified with 1× AMPure XP Beads (Beckman coulte, Brea, CA, USA) and eluted in 10 μL nuclease-free water. The purified end-prepped gDNA was then barcoded with 24 unique native barcodes using 1× Blunt/TA Ligase for 20 min at room temperature (RT). After barcoding, all the 24 samples were pooled and purified with 1× AMPure XP. After, the barcoded gDNA amplicons were eluted in 35 μL of nuclease-free water. Afterwards, 30 µL of the pooled barcoded gDNA was then ligated to the Adapter Mix II H (AMII H) using the Quick T4 Ligase for 20 min at room temperature. Purification was then performed with 1× AMPure XP using the Short Fragment Buffer (SFB), with adapter-ligated amplicons eluted in 15 μL of Elution Buffer.

##### Sequencing, Base-Calling, Alignment and SNP Detection

The prepared DNA libraries to be loaded for sequencing were prepared by thoroughly mixing 12 μL of prepared DNA library (~20 fmol), 37.5 μL of Sequencing Buffer II (SBII) and 25.5 μL of Loading Beads II (LBII). After that, 75 μL of the mixture was gently administered to the flow cells (Version FLO-MIN107) in the MinION Mk1b sequencer. Sequencing was performed between 6 and 8 h with real-time high-accuracy *guppy* base calling using the MinKNOW software (25.09.16). The resulting fastq files were processed through a custom Nextflow pipeline: *nano-rave* (Nanopore Rapid Analysis and Variant Explorer v3.1.0) [15]. After quality control (QC) checks, sequence reads were mapped to 3D7 reference sequences for each of the amplicon target genes using *minimap2 (2.31 (r1302)*. Amplicon coverage data were then generated using *BEDTools* (v2.31.1). Also, *Medaka haploid* (2.2.2) was used for variant calling to generate Variant Call Format (VCF) file outputs for each amplicon for each sample (ONT barcode), and the VCF files were processed using custom R scripts to calculate SNP frequencies at the five drug resistance loci. A cut-off of >10× coverage was applied for an amplicon to be included in the analysis.

#### 2.5.3. Data Analysis

Baseline characteristics of demographics, hematological Parameters and asymptomatic malaria infection outcomes of participants were analyzed using STATA Version 14.1 software. Descriptive statistics, chi-square test, student *t*-test, ANOVA, linear and logistic regression analysis were used to test for association between sociodemographic characteristics, hematological parameters and infection outcomes. Also, statistical significance was determined at 95% confidence interval, with a *p*-value of <0.05 considered statistically significant.

## 3. Results

### 3.1. Demographics and Clinical Characteristics of Asymptomatic Study Participants

**Figure 4:**
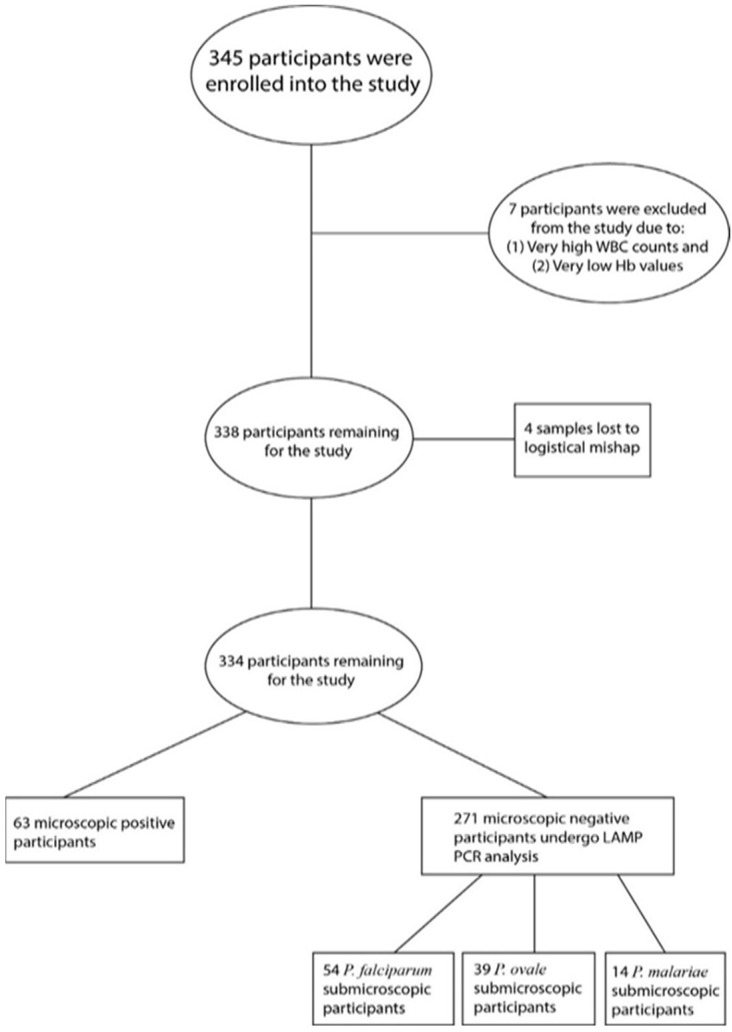
A flow diagram of the study participants.

A total of 345 participants meeting the inclusion criteria were initially enrolled. Thick and thin blood films, malaria rapid diagnostic tests (RDTs), dried blood spots for molecular analysis, and detection of drug-resistant molecular markers assessments were performed for all participants. Seven samples were excluded due to abnormally high white blood cell counts and very low hemoglobin levels, suggestive of hematological abnormalities, and four were lost during processing, leaving 334 participants for analysis (Figure 4). Participants were categorized into three groups: children (<18 years), young adults (18–35 years), and older adults (≥36 years), as summarized in Table 1.

Of the 334 participants, 196 (58.68%) were female and 138 (41.32%) were male, with a median age of 26 years (IQR: 2–79 years). RDTs detected 62 malaria-positive cases, while microscopy identified 63, with five discrepant results—two RDT-positive/microscopy-negative and three microscopy-positive/RDT-negative. All microscopy-negative samples were subsequently analyzed using loop-mediated isothermal amplification (LAMP) PCR to detect submicroscopic *Plasmodium falciparum* infections and other *Plasmodium* species, including *P. ovale* and *P. malariae*.

### 3.2. Prevalence of Asymptomatic Malaria Infections, Including Submicroscopic Infections

**Figure 5:**
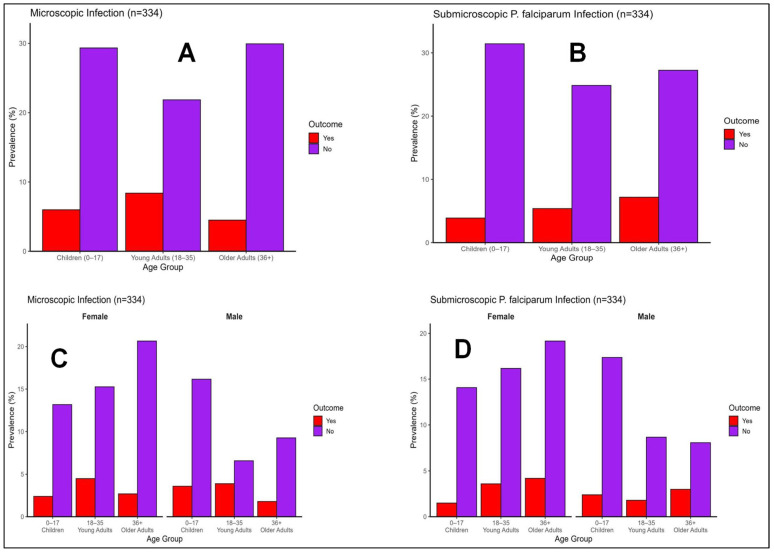
(**A**) is bar graph showing the prevalence of microscopic infections by age group. (**B**) shows the prevalence of submicroscopic infections by age group. (**C**) shows the distribution of the prevalence of microscopic infections by age groups in males and females and (**D**) shows the distribution of the prevalence of submicroscopic infections by age groups in males and females.

All baseline infections were asymptomatic, with no participants exhibiting fever or malaria-like symptoms. The overall prevalence of asymptomatic *P. falciparum* infection (microscopic and submicroscopic combined) was 35.0% (117/334). Microscopy detected 18.9% (63/334) of participants as positive for asymptomatic *P. falciparum*, while LAMP identified 16.1% (54/334) additional submicroscopic infections among the microscopy-negative cases (Figure 5A). Parasite speciation using LAMP revealed an overall 32.9% (110/334) prevalence of submicroscopic *Plasmodium* spp. infections, comprising *P. falciparum* (51.8%, 57/110), *P. ovale* (35.5%, 39/110; 11.7% overall) and *P. malariae* (12.7%, 14/110; 4.2% overall) (Figure 5B).

Age-stratified analysis showed that microscopic *P. falciparum* infection was highest among young adults (27.7%, 28/101) compared with children (16.9%, 20/118) and older adults (13.0%, 15/115), with a significant association between age and infection status (χ^2^ (2, N = 334) = 8.00, *p* = 0.018) (Figure 5C).

Submicroscopic infections were more common among older adults (20.9%, 24/115) and young adults (17.8%, 18/101) than children (12.7%, 15/118). Submicroscopic *P. ovale* infection predominated in children (16.1%, 19/118), while *P. malariae* infection was more frequent among older adults (6.1%, 7/115) (Figure 5D).

Co-infections were detected in 9 participants with *P. falciparum* and *P. ovale*, and in 2 participants each with *P. malariae* plus either *P. ovale* or *P. falciparum*. No triple-species co-infections were observed. Sex distribution showed nearly equal prevalence of microscopic infections in males (49.2%, 31/63) and females (50.8%, 32/63), while submicroscopic infections were slightly higher in females (57.4%, 31/54) than in males (48.1%, 26/54).

### 3.3. The Prevalence of Known Antimalarial Drug Resistance Genes Circulating in the Study Population

Mutations in *pfcrt*, *pfmdr1*, *pfdhfr*, *pfdhps*, and *pfk13* genes associated with resistance to chloroquine, amodiaquine, lumefantrine, pyrimethamine, sulfadoxine, and artemisinin were analyzed in 63 microscopy-positive and 54 submicroscopic *P. falciparum* samples (n = 334) using Oxford Nanopore sequencing. The prevalence of resistance-associated alleles was *pfcrt* K76T (9.5%, 95% CI: 5.1–16.9), *pfmdr1* (40%, 95% CI: 31.2–50.2), *pfdhfr* (27.1%, 95% CI: 19.3–36.7), and *pfk13* (11.4%, 95% CI: 9.8–13.3). There was *pfdhps* resistance to sulfadoxine observed in all samples. No SP-IPTp–associated mutations were detected; however, SP combination resistance occurred in 21.5% (95% CI: 14.5–30.7).

**Figure 6:**
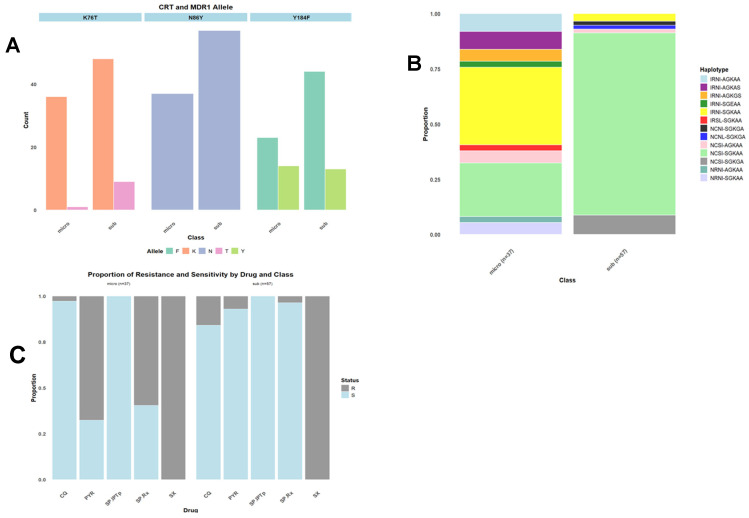
(**A**) shows the frequency distribution of CRT and MDR1 alleles in microscopic and submicroscopic infection status. (**B**) shows Plasmodium *falciparum* dhfr-dhps combined haplotypes by microscopic (micro) and sub-microscopic (sub) infection and (**C**) shows the distribution of resistance and sensitive phenotype by drug and intervention type. CQ: Chloroquine; PYR: Pyrimethamine resistance; SP.IPT_p_: Sulfadoxine-Pyrimethamine for Intermittent prevention of malaria in pregnancy; SP.RX: Sulfadoxine-Pyrimethamine resistance; SX: Sulfadoxine.

Chloroquine resistance declined markedly, with 90.5% harboring the sensitive *pfcrt* K76 allele; the K76T mutant was predominantly found in submicroscopic infections (88.9%), females (70%), and children (50%). The wild-type *pfmdr1* N86Y haplotype was absent, while the Y184F mutant (51%), associated with reduced lumefantrine susceptibility, was common, especially among microscopy-positive (52.9%) and female (46.3%) participants. Multiple *pfdhps* haplotypes were identified, led by A437G (79%), S436A (12%), A581G (9%), and A613S (5%), with no K540E, A613T, or S436F detected. For *pfdhfr*, N51I (24%), C59R (27%), and S108N (26%) were observed, with the triple mutant IRNI (35%) predominating, followed by wild-type NCSI (12%) and double mutant NRNI (4.3%). Predominant *pfdhps* genotypes included SGKAA (85.4%), SGKGA (33.1%), and AGKAS (14%), with common dhfr–dhps combinations being IRNI + SGKAA (21%), NCSI + SGKAA (10%), and IRNI + AGKAA (4.3%). Notably, *pfk13* propeller mutations linked to partial artemisinin resistance, particularly C580Y (12%), were detected exclusively in submicroscopic infections, while other variants (R539T, T573T, G453S, I601I, R575T) were not associated with clinical resistance (Figure 6).

## 4. Discussion

This study revealed a surprisingly high prevalence of asymptomatic *P. falciparum* infections (35%) among participants, a figure much higher than the reported 2% malaria prevalence for the Greater Accra Region [17]. This finding aligns with earlier reports showing that asymptomatic infections frequently exceed symptomatic ones in endemic regions [18]. Notably, other studies have shown that submicroscopic infections of Plasmodium species (32.9%) were more common than microscopic infections (18.9%), confirming that microscopy underestimates total parasite carriage and misses low-level parasitemia [19]. The high prevalence of submicroscopic infections in this low-transmission setting supports previous assertions that such infections often persist where malaria control measures have reduced overall transmission [13,20]. Similar patterns have been observed in Senegal and Tanzania [21,22], suggesting that localized hotspots and partial host immunity may sustain these reservoirs [23,24].

Age-related patterns showed that submicroscopic infections were more common among adults, consistent with evidence that partial immunity acquired over years of exposure allows adults to harbor low-level infections without symptoms [12,25]. Conversely, children with less-developed immunity, were more prone to microscopic infections. Interestingly, a notable proportion of submicroscopic *P. ovale* infections (11.5%) occurred predominantly in children, echoing findings from Ghana and other regions [26,27]. The detection of multiple species coinfections (24.1%) underscores the complexity of malaria epidemiology in low-transmission settings and the need to broaden control strategies to include non-*falciparum* infections.

In assessing diagnostic tools, the rapid diagnostic test (RDT) demonstrated high sensitivity relative to microscopy, with few discrepancies. Cases that were RDT-negative but microscopy-positive were subsequently confirmed as *P. falciparum* and *P. ovale* infections by LAMP. These discordant results may reflect a range of factors, including low parasite densities, variability in HRP2 antigen expression, or the possible presence of *pfhrp2*/*pfhrp3* gene deletions, which have been reported to affect the performance of HRP2-based RDTs [28]. As no *pfhrp2*/*pfhrp3* genotyping was performed in this study, the underlying cause of these discrepancies could not be determined. Nevertheless, the findings highlight the need for ongoing monitoring of factors that may compromise RDT performance and for continued surveillance of *pfhrp2*/*pfhrp3* deletions in malaria-endemic settings to support diagnostic accuracy. Conversely, RDT-positive but microscopy-negative cases likely represented submicroscopic infections, reaffirming the limited sensitivity of microscopy at low parasitemia.

Analysis of antimalarial drug resistance markers revealed encouraging trends and emerging concerns. The frequency of *pfcrt* K76T (9.1%) indicated a marked return of chloroquine sensitivity, likely due to reduced drug pressure following its withdrawal [29,30]. Similar reversions have been reported in Zambia and Malawi [31]. However, persistence of *pfmdr1* Y184F (51%), associated with decreased lumefantrine sensitivity, warrants attention, though clinical ACT resistance remains unreported. High mutation frequencies in *pfdhps* (A437G, S436A, A581G) and *pfdhfr* (N51I, C59R, S108N) suggest ongoing SP pressure, though no *K540E* mutation linked to IPTp failure was detected. The observed triple *pfdhfr* haplotype (IRNI, 35%) and dominant *pfdhps* haplotypes (SGKAA, SGKGA) confirm widespread SP resistance, consistent with national data [10]. Importantly, only C580Y variant of *pfk13* mutations conferring artemisinin resistance was found, though variant like R539T warrant monitoring, as has been implicated in resistance in East Africa (Rwanda, Uganda and Tanzania). Continuous genomic surveillance of both microscopic and submicroscopic infections remains essential to protect ACT efficacy. Limitations of the study include the use of a cross-sectional design, which captures only a single time-point and cannot establish causal relationships or track changes in infection status, parasite density, or resistance profiles over time. Sampling was purposive and restricted to a single tertiary hospital in Greater Accra, which may limit generalizability to community settings or other transmission zones across Ghana. While LAMP-PCR offers high sensitivity, we did not systematically screen for pfhrp2/3 deletions, which could affect the performance of HRP2-based rapid diagnostic tests. Longitudinal studies would better clarify how asymptomatic infections contribute to transmission dynamics.

## 5. Conclusions

This single-site, cross-sectional study revealed a notable hidden burden of asymptomatic and sub-microscopic *Plasmodium falciparum* infections within this specific low-transmission setting, alongside a diverse range of resistance-associated genotypes. The findings—including a return of chloroquine susceptibility, sustained resistance to sulfadoxine-pyrimethamine (SP), and the presence of emerging pfk13 variants—highlight the importance of ongoing, local drug-resistance monitoring. Within this context, strengthening molecular diagnostic capabilities and expanding targeted active surveillance through integrated control measures could support progress toward malaria elimination in this region, with findings that may inform similar low-transmission settings.

## Figures and Tables

**Table 1:** Showing the demographic and clinical characteristics of study participants.

Variables	Level	Overall (n = 334)	Anaemia n (%)		Microscopy n (%)		SubPF n (%)		SubOV n (%)		SubMA n (%)	
			Yes (n = 112)	No (n = 222)	Yes (n = 63)	No (n = 271)	Yes (n = 55)	No (n = 279)	Yes (n = 38)	No (n = 296)	Yes (n = 14)	No (n = 320)
Age Group	[0–17] (Children)	118 (35.33%)	61 (18.26%)	57 (17.07%)	20 (5.99%)	98 (29.34%)	13 (3.89%)	105 (31.44%)	18 (5.39%)	100 (29.94%)	4 (1.2%)	114 (34.13%)
	[18–35] (Young Adults)	101 (30.24%)	27 (8.08%)	74 (22.16%)	28 (8.38%)	73 (21.86%)	18 (5.39%)	83 (24.85%)	8 (2.4%)	93 (27.84%)	3 (0.9%)	98 (29.34%)
	[36+] (Older Adults)	115 (34.43%)	24 (7.19%)	91 (27.25%)	15 (4.49%)	100 (29.94%)	24 (7.19%)	91 (27.25%)	12 (3.59%)	103 (30.84%)	7 (2.1%)	108 (32.34%)
Sex	FEMALE	196 (58.68%)	62 (18.56%)	134 (40.12%)	32 (9.58%)	164 (49.1%)	31 (9.28%)	165 (49.4%)	19 (5.69%)	177 (52.99%)	10 (2.99%)	186 (55.69%)
	MALE	138 (41.32%)	50 (14.97%)	88 (26.35%)	31 (9.28%)	107 (32.04%)	24 (7.19%)	114 (34.13%)	19 (5.69%)	119 (35.63%)	4 (1.2%)	134 (40.12%)
Education	BASIC	68 (20.36%)	28 (8.38%)	40 (11.98%)	11 (3.29%)	57 (17.07%)	9 (2.69%)	59 (17.66%)	10 (2.99%)	58 (17.37%)	2 (0.6%)	66 (19.76%)
	N/A	52 (15.57%)	33 (9.88%)	19 (5.69%)	6 (1.8%)	46 (13.77%)	6 (1.8%)	46 (13.77%)	8 (2.4%)	44 (13.17%)	2 (0.6%)	50 (14.97%)
	SECONDARY	127 (38.02%)	33 (9.88%)	94 (28.14%)	30 (8.98%)	97 (29.04%)	24 (7.19%)	103 (30.84%)	12 (3.59%)	115 (34.43%)	7 (2.1%)	120 (35.93%)
	TERTIARY	87 (26.05%)	18 (5.39%)	69 (20.66%)	16 (4.79%)	71 (21.26%)	16 (4.79%)	71 (21.26%)	8 (2.4%)	79 (23.65%)	3 (0.9%)	84 (25.15%)
Occupation	N/A	57 (17.07%)	36 (10.78%)	21 (6.29%)	8 (2.4%)	49 (14.67%)	7 (2.1%)	50 (14.97%)	9 (2.69%)	48 (14.37%)	2 (0.6%)	55 (16.47%)
	PRIVATE SECTOR	130 (38.92%)	34 (10.18%)	96 (28.74%)	23 (6.89%)	107 (32.04%)	27 (8.08%)	103 (30.84%)	12 (3.59%)	118 (35.33%)	8 (2.4%)	122 (36.53%)
	PUBLIC SECTOR	30 (8.98%)	3 (0.9%)	27 (8.08%)	2 (0.6%)	28 (8.38%)	8 (2.4%)	22 (6.59%)	4 (1.2%)	26 (7.78%)	2 (0.6%)	28 (8.38%)
	RETIRED	27 (8.08%)	5 (1.5%)	22 (6.59%)	4 (1.2%)	23 (6.89%)	4 (1.2%)	23 (6.89%)	1 (0.3%)	26 (7.78%)	0 (0%)	27 (8.08%)
	STUDENT	90 (26.95%)	34 (10.18%)	56 (16.77%)	26 (7.78%)	64 (19.16%)	9 (2.69%)	81 (24.25%)	12 (3.59%)	78 (23.35%)	2 (0.6%)	88 (26.35%)

## Data Availability

The data presented in this study are available on request from the corresponding author due to ethical reasons.

## Supplementary Materials

The following supporting information can be downloaded at: https://www.mdpi.com/article/10.3390/tropicalmed11070190/s1, Supplementary material: Plasmodium falciparum Species-Specific Primers–Multiplex PCR, Primers used in LAMP assay, Loop-Mediated Isothermal Amplification (LAMP) Protocol Reagent Table and Multiplex PCR Protocol Reagent Table.
