# Supplementary material for: Submicroscopic Plasmodium falciparum Carriage and Molecular Markers of Antimalarial Drug Resistance Among Outpatients Attending Korle Bu Teaching Hospital, Ghana
Source: Trop Med Infect Dis. 2026 Jul 9;11(7):190. doi: 10.3390/tropicalmed11070190 (PMC13417555; doi:10.3390/tropicalmed11070190)
Supplement: Supplementary file 1 [file tropicalmed-11-00190-s001.zip › tropicalmed-4344272-supplementary.pdf]

Plasmodium falciparum Species-Specific Primers – Multiplex PCR

🧬 Gold Standard: 18S rRNA (Nested PCR, Snounou protocol)

Most common for diagnosis & research

- **Primary PCR (genus-level):**
  rPLU1: `5'-TCAAAGATTAAGCCATGCAAGTGA-3'`
  rPLU5: `5'-CCTGTTGTTGCCTTAAACTTC-3'`
  Product: ~620 bp
- **Secondary PCR (P. falciparum-specific):**
  FAL1: `5'-TTAAGGATGCAGAGCTAATAC-3'`
  FAL2: `5'-AAGATTTACGATCAACTGTC-3'`
  Product: ~205 bp
  Annealing: 58°C

🧬 Single-Step PCR (18S rRNA)

Direct, no nested step

- Forward: `5'-GTGTGTATCAATCGAGTTTC-3'`
- Reverse: `5'-ACGATCGTTTTCTGCCGTTG-3'`
- Product: ~130 bp
- Specificity: No cross-amplification with P. vivax, P. malariae, P. ovale, P. knowlesi

🧬 High-Sensitivity: Mitochondrial COX1 (multi-copy)

More sensitive for low parasitemia

- Pf-COX1-F: `5'-ATGGCAGTATTCATTTGATTTG-3'`
- Pf-COX1-R: `5'-TCTCCATTTCCATTTGTTTGT-3'`
- Product: ~350 bp
- Annealing: 56°C

🧬 qPCR Primers + Probe (18S rRNA)

For quantification & high-throughput

- Forward: `5'-CTTTTGAGAGTTTTGTTCTGG-3'`
- Reverse: `5'-TAAGGAATGTTTTCGCAAG-3'`
- Probe: `5'-FAM-ATTGGTTTTGACGTTTTAATTTT-TAMRA-3'`
- Limit of detection: ~0.3 parasites/µL

🧬 Alternative: PfHRP2 Gene

Species-unique marker

- Forward: `5'-GCAGAAGGTTTTGCACATGC-3'`
- Reverse: `5'-GTGGATGTGGTGCCTGTTTG-3'`
- Product: ~280 bp

Note: Avoid in regions with widespread pfhrp2/3 deletions

Primers used in LAMP

The Species-specific LAMP primer sets consisted of six primers: the forward outer primer (F3), backward outer primer (B3), forward inner primer (FIP), backward inner primer (BIP), loop forward primer (LF), and loop backward primer (LB). Primer sequences for *P. falciparum*, *P. malariae*, and *P. ovale* are provided below.

F3: `5' - GGGAAACTTGTGCCCTTTG - 3'`

B3: `5' - CAATTGGTTTTGACCATTTTAG - 3'`

FIP: `5' - TGGCATGGAAGTCACACCG - TTTT - GATCTTCTTCGATTTTTGGC - 3'`
(F1c - TTTT linker - F2)

BIP: `5' - TGGTTTTGTTGGCCTGTTGG - TTTT - CCGTCTTGAGAGAGCAGT - 3'`
(B1c - TTTT linker - B2)

LF (Loop Forward): `5' - TGCTGCTGTGTACAGTGCT - 3'`

LB (Loop Backward): `5' - TGCTGGTGCATGTAGTTGAG - 3'`

Loop-Mediated Isothermal Amplification (LAMP) Protocol Reagent Table

Reactions were prepared on ice to prevent premature amplification:

**Component**	**Volume**	**Final Concentration**
Nuclease-free water	Up to 25 µL	—
10× Isothermal Amplification Buffer	2.5 µL	1×
50 mM $MgSO_4$*	1.0–3.0 µL	4–8 mM
10 mM dNTP mix	3.5–4.0 µL	1.4–1.6 mM each
5 M Betaine (optional)	5.0 µL	1 M
F3 + B3 primers (10 µM each)	0.5 µL each	0.2 µM each
FIP + BIP primers (40 µM each)	1.0 µL each	1.6 µM each
LF + LB primers (20 µM each, optional)	0.5–1.0 µL each	0.4–0.8 µM each
Template DNA	1.0–5.0 µL	1–500 ng
Bst DNA Polymerase (8 U/µL)	1.0–2.0 µL	8–16 U
Detection dye	0.5 µL	As recommended

Multiplex PCR Protocol Reagent Table

Prepared on ice; include no-template control and positive control:

**Component**	**Volume**	**Final Concentration**
Nuclease-free water	Up to 25 µL	—
10× Multiplex PCR Buffer	2.5 µL	1×
25 mM $MgCl_2$*	1.5–4.0 µL	1.5–4.0 mM
10 mM dNTP mix	0.5 µL	0.2 mM each
Primer mix (all pairs combined)	1.0–3.0 µL	0.1–0.5 µM each primer
Enhancer mix (optional)	1.0–2.5 µL	As recommended
Template DNA	1.0–5.0 µL	10–200 ng
Hot-start Taq Polymerase (5 U/µL)	0.25–0.5 µL	1.25–2.5 U
